# Structural neuroimaging measures and lifetime depression across levels of phenotyping in UK biobank

**DOI:** 10.1038/s41398-022-01926-w

**Published:** 2022-04-13

**Authors:** Mathew A. Harris, Simon R. Cox, Laura de Nooij, Miruna C. Barbu, Mark J. Adams, Xueyi Shen, Ian J. Deary, Stephen M. Lawrie, Andrew M. McIntosh, Heather C. Whalley

**Affiliations:** 1 Division of Psychiatry, University of Edinburgh, Edinburgh, UK; 2 Department of Psychology, University of Edinburgh, Edinburgh, UK

**Keywords:** Neuroscience, Psychiatric disorders

## Abstract

Depression is assessed in various ways in research, with large population studies often relying on minimal phenotyping. Genetic results suggest clinical diagnoses and self-report measures of depression show some core similarities, but also important differences. It is not yet clear how neuroimaging associations depend on levels of phenotyping. We studied 39,300 UK Biobank imaging participants (20,701 female; aged 44.6 to 82.3 years, M = 64.1, SD = 7.5) with structural neuroimaging and lifetime depression data. Past depression phenotypes included a single-item self-report measure, an intermediate measure of ‘probable’ lifetime depression, derived from multiple questionnaire items relevant to a history of depression, and a retrospective clinical diagnosis according to DSM-IV criteria. We tested (i) associations between brain structural measures and each depression phenotype, and (ii) effects of phenotype on these associations. Depression-brain structure associations were small (β < 0.1) for all phenotypes, but still significant after FDR correction for many regional metrics. Lifetime depression was consistently associated with reduced white matter integrity across phenotypes. Cortical thickness showed negative associations with Self-reported Depression in particular. Phenotype effects were small across most metrics, but significant for cortical thickness in most regions. We report consistent effects of lifetime depression in brain structural measures, including reduced integrity of thalamic radiations and association fibres. We also observed significant differences in associations with cortical thickness across depression phenotypes. Although these results did not relate to level of phenotyping as expected, effects of phenotype definition are still an important consideration for future depression research.

## Introduction

Major depressive disorder (MDD) affects a substantial and increasing proportion of the population [1, 2]. There are currently no objective biomarkers to guide diagnosis, which instead relies on the time-consuming subjective assessment of a range of possible symptoms [3]. Several diagnostic tools are available, along with a variety of assessments used for research purposes. The range of available measures all differ at least slightly in terms of how they define depression, yet much research uses these various definitions interchangeably. This may underlie inconsistencies across studies, as studying different definitions of depression could mean focusing on quite different aspects of the disorder. In the context of the current move towards larger community-based studies, detailed face-to-face clinical assessments are often impractical, with simpler self-report and questionnaire-based measures being favoured instead. The implications for the field of depression research are not yet clear, and better understanding of the impact on results of different definitions and levels of phenotyping is imperative.

Howard et al. [4] recently studied genetic contributions to lifetime depression, including both current and past cases, in a very large sample of over 320,000 UK Biobank participants. Importantly, the study assessed genome-wide associations for multiple definitions of lifetime depression based on: (i) a broad classification derived from minimal phenotyping of self-reported treatment seeking for ‘nerves, anxiety or depression’, (ii) an intermediate phenotype of ‘probable’ depression derived from responses to several self-report mental health questionnaire items, and (iii) a clinical MDD phenotype derived from diagnosis by doctors while in hospital. Interestingly, these three different phenotypes all showed similar genetic associations to one another and to previous results, suggesting that each related to an overlapping core component of depression. However, there were also a number of significant associations specific to each phenotype, suggesting that different definitions reflect slightly different underlying constructs, with some differences in genetic basis. In another study of the same sample, Cai et al. [5] found that depression heritability estimates and genetic associations also varied according to level of phenotyping, with single-item self-report measures of lifetime depression more closely related to neuroticism than to a past clinical diagnosis of MDD. We hypothesised that similar differences in relation to depth of phenotyping would be observed for associations with structural neuroimaging measures.

A considerable amount of research has focused on associations between depression and neuroimaging-derived measures of the brain. Depression-related structural differences have been observed in a range of areas [6–8], but the strongest differences are often volumetric reductions in the hippocampus [9, 10] and regions of prefrontal cortex [11, 12]. Some studies have shown increases in volumes of certain regions, such as amygdala [13, 14] and anterior cingulate cortex [15], although these may represent confounding effects of medication rather than depression itself [16]. The use of various definitions of depression may also account for some of the variability in results across studies. Measures of white matter integrity also show associations with depression [17, 18], indicating reduced connectivity, particularly between frontal and limbic regions [19, 20]. Functional neuroimaging findings provide further support for reduced connectivity in depression [21–23], which would be consistent with white matter degeneration. It is essential to address the potential impact of variable and often coarse assessment of MDD, especially given the phenotypic and genetic evidence that different definitions may reflect partially separable constructs.

We therefore sought to explicitly test the effects of lifetime depression phenotyping on associations with structural neuroimaging features. The present study used definitions at three phenotyping levels, and both grey and white matter structural metrics for UK Biobank imaging participants. We tested (i) lifetime depression associations with measures of cortical regions, subcortical structures and white matter tracts for each phenotype, and (ii) the effects of phenotype definition on these associations. We expected to find (i) reductions in grey matter size and white matter integrity among depressed subjects in a range of areas described above, and (ii) that associations between lifetime depression and structural brain metrics would vary considerably across levels of phenotyping, with greater effect sizes for more in-depth phenotypes. These results would inform on whether associations between depression and neuroimaging measures depend on how depression is phenotyped or defined, relevant for future large-scale studies of the disorder.

## Methods

### Participants

UK Biobank (http://www.ukbiobank.ac.uk) includes data on 503,325 members of the general UK population, recruited between 2006 and 2010 [24]. Participants originally provided information on a wide range of health, lifestyle, environment and other variables, and have provided further data at subsequent follow-ups. The present study focused on the first 39,300 of these participants for whom structural neuroimaging data were made available. Participants with severe neuropsychological disorders (including schizophrenia, dementia, Parkinson’s disease and multiple sclerosis) were excluded from all analyses due to the marked effects that such disorders can have on brain structural metrics. The number of remaining subjects who also provided data on lifetime depression ranged from 15,079 for Probable Depression to 26,875 for Self-reported Depression. Descriptives are reported in Table 1.

**Table 1:** Descriptive statistics for each lifetime depression phenotype.

	Self-reported Depression	Probable Depression	CIDI-assessed MDD
Subjects (N, %)	26,875 (68.4)	15,079 (38.4)	19,430 (49.4)
Age (years; M, SD)	64.3 (7.6)	63.3 (7.5)	64.2 (7.5)
Sex			
Males (N, %)	13,977 (52.0)	7230 (47.9)	9165 (47.2)
Females (N, %)	12,898 (48.0)	7849 (52.1)	10,265 (52.8)
Depression			
Cases (N, %)	2561 (9.5)	6787 (45.0)	6456 (33.2)
Controls (N, %)	24,314 (90.5)	8292 (55.0)	12,974 (66.8)
Neuroticism score (M, SD)	3.3 (3.0)	3.8 (3.2)	3.4 (3.1)
Global CT (mm; M, SD)	2.67 (0.11)	2.67 (0.11)	2.67 (0.11)
Total CSA (mm^2^; M, SD)	169,880 (15,409)	169,498 (15,308)	169,399 (15,276)
Total CV (mm^3^; M, SD)	500,279 (47,867)	499,502 (47,575)	498,866 (47,143)
Total SCV (mm^3^; M, SD)	188,547 (169,19)	188,073 (16,894)	187,912 (16,691)
Global FA (M, SD)	0.011 (1.002)	0.030 (0.990)	0.016 (0.992)
Global MD (M, SD)	0.018 (1.003)	–0.002 (0.988)	–0.001 (0.992)

*CIDI* composite International Diagnostic Interview (short form), *MDD* Major Depressive Disorder, *CT* cortical thickness, *CSA* cortical surface area, *CV* cortical volume, *SCV* subcortical volume, *FA* fractional anisotropy, *MD* mean diffusivity. Descriptive statistics for secondary phenotypes are reported in Supplementary Materials (Table S1).

### Depression phenotypes

As the aim of this study was to assess the effect of different definitions of lifetime depression on underlying imaging features, history of depression was phenotyped in multiple ways. We focused primarily on three of the five definitions assessed previously by Cai et al. [5]: a minimal phenotype, Self-reported Depression; an intermediate phenotype, Probable Depression; and a clinical diagnosis, CIDI-assessed MDD. These phenotypes are described in further detail below, and numbers of cases and controls included by each definition are reported in Table 1. The same details for secondary phenotypes are also included in Supplementary Materials (Table S1).

#### Minimal phenotypes

Our primary minimal phenotype, Self-reported Depression, was based on whether participants did (cases) or did not (controls) specifically report a current or past diagnosis of depression when asked to provide details of any physical or mental disorders they had ever been diagnosed with. A second simple measure, Self-reported Treatment, is also reported in Supplementary Materials.

#### Intermediate phenotypes

An approximate measure of lifetime depression was previously created for UK Biobank participants [25], in lieu of more reliable clinical assessments. This was derived from relevant questions in an informal mental health questionnaire, administered at each UK Biobank assessment by touchscreen. Questions relevant to depression included those on incidence and duration of previous episodes of low mood or anhedonia, as well as past treatment by a general practitioner or psychiatrist. As described previously, Smith et al. [25] combined responses to these items into measures of ‘probable single episode’, ‘probable mild recurrent’ and ‘probable severe recurrent’. We identified those who strictly met all criteria for any of these categories as cases of Probable Depression. Probable Depression controls were those who met none of the criteria. Cai et al. [5] used this definition as another minimal phenotype, but we present it as intermediate, being more detailed than single-item measures, but less thorough than clinical assessments. A closely related second intermediate phenotype, Recurrent Depression, is included in Supplementary Materials.

#### Clinical phenotypes

As part of an online assessment (introduced after imaging assessments had begun), some UK Biobank participants completed the short form of the Composite International Diagnostic Interview (CIDI-SF) [26]. Although not strictly a clinical assessment, the CIDI-SF is based on DSM-IV criteria. The section on MDD symptomatology provided a score of between zero and seven. Those with a score of five or more were classified as cases, as this indicated past symptomatology consistent with a diagnosis of MDD. However, as the assessment was completed up to 2.7 years after the imaging assessment, CIDI-assessed MDD cases who did not report any previous depressive symptoms at the time of the imaging assessment were excluded, as were controls who had previously reported symptoms. ICD-diagnosed MDD is included in Supplementary Materials as a secondary clinical phenotype.

### Brain imaging data

Brain magnetic resonance imaging (MRI) data were acquired at two sites, but each with the same Siemens (Berlin/Munich, Germany) Skyra 3 T scanner and 32-channel head coil, and using the same protocol, as described previously [27]. The present study used T1-weighted images, acquired using a 3D magnetisation-prepared rapid-acquisition gradient-echo (MPRAGE) sequence at 1 mm isotropic resolution, and diffusion-weighted images, acquired using a reverse phase-encoded fat-saturation sequence at 2 mm isotropic resolution and modelled by the diffusion tensor.

Cortical measures were derived from raw T1 images by UK Biobank [28] using FreeSurfer version 6.0 [29–31]. For each T1, the brain was segmented into grey matter, white matter and cerebrospinal fluid, and grey matter further segmented into cortical and subcortical regions. The cortex was then divided into 34 regions per hemisphere, according to the Desikan-Killany atlas [32]. Output was visually assessed for errors, and participants with any major errors in segmentation or cortical parcellation were excluded. For each of the 68 cortical regions, UK Biobank provided measures of mean thickness, surface area and volume. Prior to analysis, we summed (cortical surface area, cortical volume) or weighted-averaged (cortical thickness) metrics for some smaller regions, producing measures for 23 regions of interest per hemisphere, as detailed in Supplementary Materials. These were further combined to produce global and lobar (frontal, parietal, temporal, occipital and cingulate) measures.

The volumes of seven subcortical structures per hemisphere and diffusion measures – fractional anisotropy (FA) and mean diffusivity (MD) – of 27 tracts per hemisphere were previously derived using FSL by the UK Biobank imaging team [27, 28]. Subcortical volumes were summed to produce a measure of overall subcortical volume. Tract-type (association fibres, thalamic radiations and projection fibres) and global summary measures of FA and MD were derived by principal components analysis, using scores on the first unrotated component (detailed in Supplementary Materials). Coordinates of head position within the scanner were provided by UK Biobank to be included as covariates in analyses, along with other covariates described below. Whole-brain volume (WBV) was also included as a covariate in order to reduce the effects of individual and phenotype differences in overall brain size on region-wise associations.

### Statistical analysis

Data were analysed primarily in R version 3.2.3 [33]. Firstly, as an additional quality control step, outliers – defined as further than three standard deviations from the mean – were removed from all neuroimaging measures. We then assessed (i) main effects of lifetime depression case-control status on each regional cortical metric, subcortical volume and white matter tract measure, (ii) differences between depression phenotypes in each of these effects, and (iii) second-level effects of phenotype on associations across all regions/tracts for each metric. Effects of case-control status were assessed using linear mixed models, including both left and right metrics as repeated measures, and controlling age, age^2^, sex, site, scanner head position coordinates and WBV in each model. Effects of phenotype definition were then assessed using z-tests to test for significant range in β coefficients across definitions. This meant testing differences between β coefficients for the two phenotypes that showed the greatest disparity in associations with each measure, automatically accounting for any differences between phenotypes in number of included participants. FDR correction was applied to *p* values across all regions and phenotypes for each metric separately. Results are reported in terms of standardised β coefficients, with *p* values and *q* values (FDR-corrected *p* values) below .05 considered as significant. A diagram summarising the main analyses performed is included in Supplementary Materials (Fig. S1).

## Results

Descriptive statistics and differences between lifetime depression definitions in key variables are reported in Table 1. As above, Ns ranged from 15,079 to 26,875, due to differences in availability of data and in criteria. Proportion of cases ranged more widely, from 9.5% for Self-reported Depression to 45.0% for Probable Depression (ANOVA F_1,3_ = 118.3, *p* < 0.001)—although this was largely due to a difference between phenotypes in exclusion of controls. Mean age ranged by just one year across definitions, from 63.3 years for Probable Depression to 64.3 years for Self-reported Depression, but this was enough to produce a significant effect (F_1,3_ = 89.8, *p* < 0.001). Overall, 47.4% of subjects were male and 52.6% were female, but there was a small amount of variability in this proportion across definitions (F_1,3_ = 62.3, *p* < 0.001). There was also a small but still significant effect of depression phenotype on whole-brain volume (F_1,3_ = 31.8, *p* = 0.011). Age, sex and WBV were therefore included as controls in subsequent regression models. Secondary phenotypes – Self-reported Treatment, Recurrent Depression, ICD-diagnosed MDD and Neuroticism – are summarised in Supplementary Materials (Table S1).

### Global, lobar and tract-type measures

**Fig. 1:**
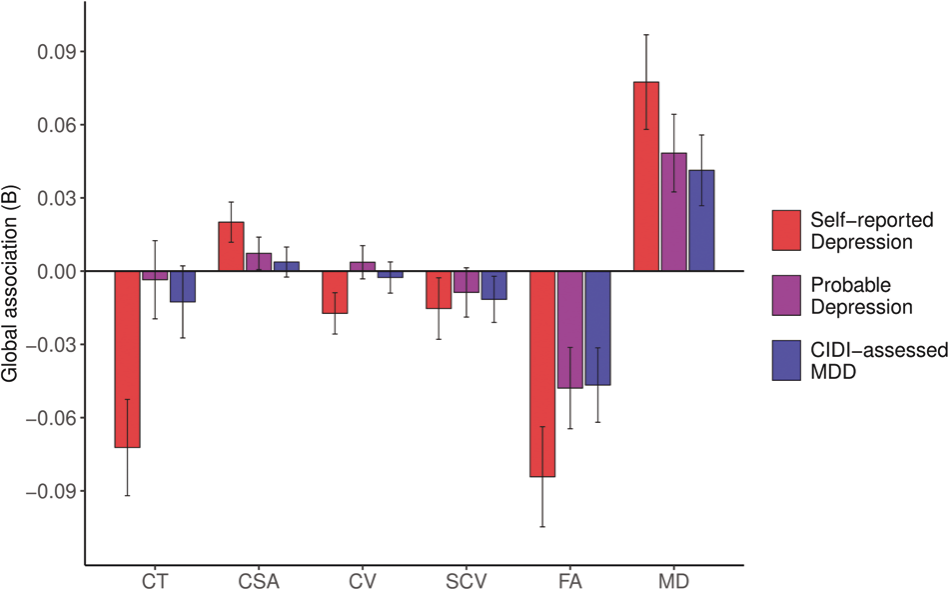
Associations between lifetime depression phenotypes and global structural brain metrics. *Notes*. CIDI = Composite International Diagnostic Interview (short form); MDD = Major Depressive Disorder; CT = global mean cortical thickness; CSA = total cortical surface area; CV = total cortical volume; SCV = total subcortical volume; FA = global mean fractional anisotropy; MD = global mean diffusivity. Bars represent β coefficients for global metric associations with Self-reported Depression (red), Probable Depression (purple) and CIDI-assessed MDD (blue); error bars represent the standard error of the estimated coefficient.

Linear modelling estimates of associations between current or past depression and global brain structural measures are shown in Fig. 1. Self-reported Depression was significantly associated with lower global mean cortical thickness (β = –0.072, SE = 0.020, *p* < 0.001), greater total cortical surface area (β = 0.020, SE = 0.008, *p* = 0.015) and lower total cortical volume (β = –0.017, SE = 0.008, *p* = 0.041), as well as with lower global FA (β = –0.084, SE = 0.021, *p* < 0.001) and higher global MD (β = 0.077, SE = 0.019, *p* < 0.001). Probable Depression and CIDI-assessed MDD were also both associated with lower global FA (β = –0.048, SE = 0.017, *p* = 0.004; β = –0.047, SE = 0.015, *p* = 0.002) and higher global MD (β = 0.048, SE = 0.016, *p* = 0.002; β = 0.041, SE = 0.014, *p* = 0.004).

Associations with lobar and tract-type summary measures, also estimated by linear modelling, are reported in Table 2. Self-reported Depression and CIDI-assessed MDD both showed significant negative associations with most lobar cortical thickness measures (Table 2), while Probable Depression (β = 0.045, SE = 0.016, *q* = 0.019) and CIDI-assessed MDD (β = 0.047, SE = 0.015, *q* = 0.011) both associated with greater cortical thickness specifically in the occipital lobe. Probable Depression also showed a significant association with greater cingulate cortical surface area (β = 0.045, SE = 0.012, *q* = 0.012).

**Table 2:** Associations between lifetime depression phenotypes and structural metrics for the whole brain, cortical lobes and white matter tract types.

	Self-reported Depression (β)	Probable Depression (β)	CIDI-assessed MDD (β)	Phenotype effect (z)
Global CT	–0.072***	–0.004	–0.013	0.069**
Frontal lobe CT	–0.100***	–0.028	–0.036*	0.072**
Parietal lobe CT	–0.052**	0.007	0.011	0.063**
Temporal lobe CT	–0.088***	–0.005	–0.034*	0.083**
Occipital lobe CT	0.030	0.045**	0.047**	0.017
Cingulate CT	–0.102***	–0.019	–0.035*	0.083**
Total CSA	0.020*	0.007	0.004	0.016
Frontal lobe CSA	0.029**	0.015	0.008	0.021
Parietal lobe CSA	0.013	–0.001	–0.008	0.021
Temporal lobe CSA	0.009	0.012	0.005	0.007
Occipital lobe CSA	0.003	–0.008	–0.004	0.011
Cingulate CSA	0.045***	0.019	0.026**	0.026
Total CV	–0.017*	0.004	–0.003	0.021
Frontal lobe CV	–0.021*	0.001	–0.008	0.021
Parietal lobe CV	–0.02	–0.001	–0.007	0.019
Temporal lobe CV	–0.029**	0.007	–0.007	0.035**
Occipital lobe CV	0.019	0.014	0.019	0.006
Cingulate CV	0.004	0.011	0.009	0.008
Total SCV	–0.015	–0.009	–0.012	0.007
Global FA	–0.084***	–0.048**	–0.047**	0.038
Association fibres FA	–0.083***	–0.045**	–0.041**	0.042
Thalamic radiations FA	–0.092***	–0.047**	–0.066***	0.045
Projection fibres FA	–0.014	–0.045**	–0.003	0.041
Global MD	0.077***	0.048**	0.041**	0.036
Association fibres MD	0.054**	0.027	0.025	0.029
Thalamic radiations MD	0.092***	0.067***	0.057***	0.035
Projection fibres MD	0.061**	0.041*	0.041**	0.019

Phenotype effects for each metric represent the difference, assessed using z-tests, between the two most disparate of the β coefficients for the three phenotypes’ associations with that metric. *, ** and *** represent significant results at *p* < 0.05, *p* < 0.01 and *p* < 0.001, respectively; significant results after FDR correction are highlighted in bold. Corresponding structural metric associations for secondary phenotypes are reported in Supplementary Materials (Table S2).

*CIDI* composite International Diagnostic Interview (short form), *MDD* Major Depressive Disorder, *CT* cortical thickness, *CSA* cortical surface area, *CV* cortical volume, *SCV* subcortical volume, *FA* fractional anisotropy, *MD* mean diffusivity.

White matter tract types generally showed the same positive associations for FA and negative associations for MD with all lifetime depression phenotypes, although FA in projection fibres showed weaker associations with Self-reported Depression and CIDI-assessed MDD, while MD in association fibres showed weaker associations with Probable Depression and CIDI-assessed MDD (Table 2).

As the association with global mean cortical thickness was stronger for Self-reported Depression than for the other two phenotypes, there was a significant effect of phenotype definition on associations with this metric (z = 0.069, SE = 0.025, *p* = 0.007). There were also significant effects of phenotype definition on associations with cortical thickness in frontal (z = 0.072, SE = 0.025, *q* = 0.016), parietal (z = 0.063, SE = 0.024, *q* = 0.025) and temporal (z = 0.083, SE = 0.026, *q* = 0.011) lobes, as well as cingulate cortex (z = 0.083, SE = 0.026, *q* = 0.011). There was a nominally significant effect of phenotype definition on cortical volume associations in the temporal lobe (z = 0.035, SE = 0.013, *p* = 0.008), but this did not survive correction for multiple comparisons.

As reported in Supplementary Materials, the three secondary lifetime depression phenotypes showed similar negative associations with cortical thickness and FA, and similar positive associations with MD and some occipital and cingulate cortex measures, while Neuroticism only associated significantly with total cortical surface area (Table S2).

### Individual regions and tracts

**Fig. 2:**
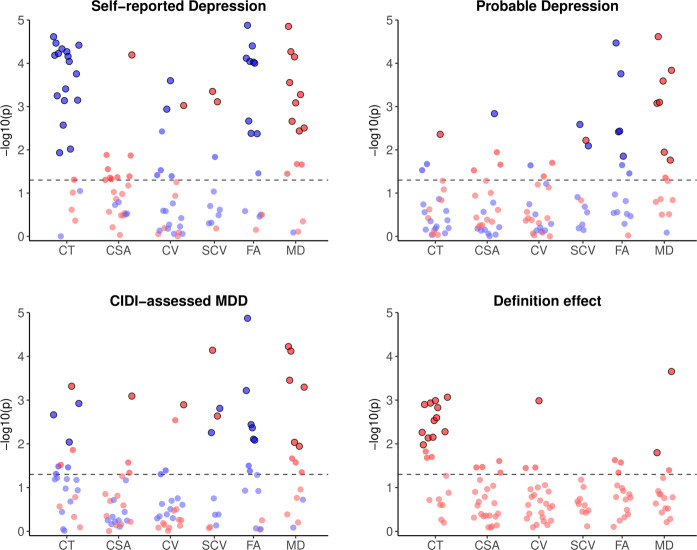
Significance of associations between lifetime depression phenotypes and individual cortical, subcortical and white matter metrics, and effects of phenotype definition on these associations. *Notes*. CIDI = Composite International Diagnostic Interview (short form); MDD = Major Depressive Disorder; CT = cortical thickness; CSA = cortical surface area; CV = cortical volume; SCV = subcortical volume; FA = fractional anisotropy; MD = mean diffusivity. Phenotype effects for each metric represent the difference, assessed using z-tests, between the two most disparate of the β coefficients for the three phenotypes’ associations with that metric. Red and blue points represent positive and negative associations, respectively. Horizontal dashed lines represent *p* = 0.05; outlined points represent associations that remained significant following FDR correction. β coefficients for all plotted results are reported in Supplementary Materials (Tables S3–S8).

Lifetime depression associations for all individual cortical regions, subcortical volumes and white matter tracts are illustrated in Fig. 2. After FDR correction, Self-reported Depression showed significant associations with lower cortical thickness in 17 of 23 regions, with the strongest associations in superior (β = –0.096, SE = 0.018, *q* < 0.001) and inferior (β = –0.093, SE = 0.018, *q* < 0.001) frontal gyri. Self-reported Depression also showed significant associations with higher surface area (β = 0.057, SE = 0.013, *q* = 0.002) and volume (β = 0.047, SE = 0.014, *q* = 0.034) of the cingulate isthmus, lower volume of inferior parietal (β = –0.038, SE = 0.012, *q* = 0.034) and middle temporal (β = –0.047, SE = 0.013, *q* = 0.023) cortices, and greater volume of the caudate nucleus (β = 0.060, SE = 0.017, *q* = 0.009) and putamen (β = 0.054, SE = 0.016, *q* = 0.010). Further, Self-reported Depression showed consistently negative associations with white matter FA, particularly in posterior thalamic radiations (β = –0.115, SE = 0.019, *q* < 0.001) and the forceps minor (β = –0.100, SE = 0.020, *q* < 0.001), and consistently positive associations with MD, strongest in anterior thalamic radiations (β = 0.105, SE = 0.018, *q* < 0.001) and, again, forceps minor (β = 0.087, SE = 0.020, *q* < 0.001).

Probable Depression showed weaker associations with cortical metrics, with only greater lateral occipital cortical thickness (β = 0.043, SE = 0.015, *q* = 0.025) and lower pericalcarine cortical surface area (β = –0.046, SE = 0.014, *q* = 0.045) remaining significant after correcting for multiple comparisons. Associations with lower brainstem grey matter volume (β = –0.034, SE = 0.011, *q* = 0.018), smaller ventral diencephalon (β = –0.027, SE = 0.010, *q* = 0.040) and larger putamen (β = 0.035, SE = 0.013, *q* = 0.034) also remained significant after FDR correction. As for Self-reported Depression, Probable Depression showed many negative associations with FA and positive associations with MD in white matter tracts, both strongest in posterior thalamic radiations (β = –0.080, SE = 0.016, *q* < 0.001; β = 0.080, SE = 0.015, *q* < 0.001) and the forceps minor (β = –0.063, SE = 0.017, *q* = 0.002; β = 0.060, SE = 0.017, *q* = 0.002).

Following FDR correction, CIDI-assessed MDD showed significant negative associations with thickness of superior frontal (β = -0.041, SE = 0.013, *q* = 0.016), fusiform (β = –0.035, SE = 0.013, *q* = 0.041) and posterior cingulate (β = –0.042, SE = 0.013, *q* = 0.012) cortices, and positive associations with lateral occipital cortical thickness (β = 0.048, SE = 0.014, *q* = 0.007) and cingulate isthmus surface area (β = 0.032, SE = 0.010, *q* = 0.030) and volume (β = 0.034, SE = 0.011, *q* = 0.034). CIDI-assessed MDD also showed significant associations with lower brainstem grey matter (β = –0.029, SE = 0.011, *q* = 0.034) and ventral diencephalon (β = –0.030, SE = 0.010, *q* = 0.014) volumes, but greater volumes of caudate nucleus (β = 0.055, SE = 0.013, *q* = 0.002) and putamen (β = 0.036, SE = 0.012, *q* = 0.018). Consistent with other phenotypes, CIDI-assessed MDD showed negative associations with FA in most white matter tracts, particularly posterior (β = –0.085, SE = 0.014, *q* < 0.001) and anterior (β = –0.050, SE = 0.015, *q* = 0.005) thalamic radiations, and generally positive associations with MD, strongest in anterior (β = 0.060, SE = 0.013, *q* < 0.001) and superior (β = 0.057, SE = 0.013, *q* < 0.001) thalamic radiations.

Associations with lower FA and higher MD were also consistent across the secondary phenotypes of lifetime depression (although not Neuroticism), while Self-reported Treatment and ICD-diagnosed MDD, as for Self-reported Depression, showed a number of significant associations with lower cortical thickness (Fig. S2; Tables S9–S14).

### Phenotype definition effects

**Fig. 3:**
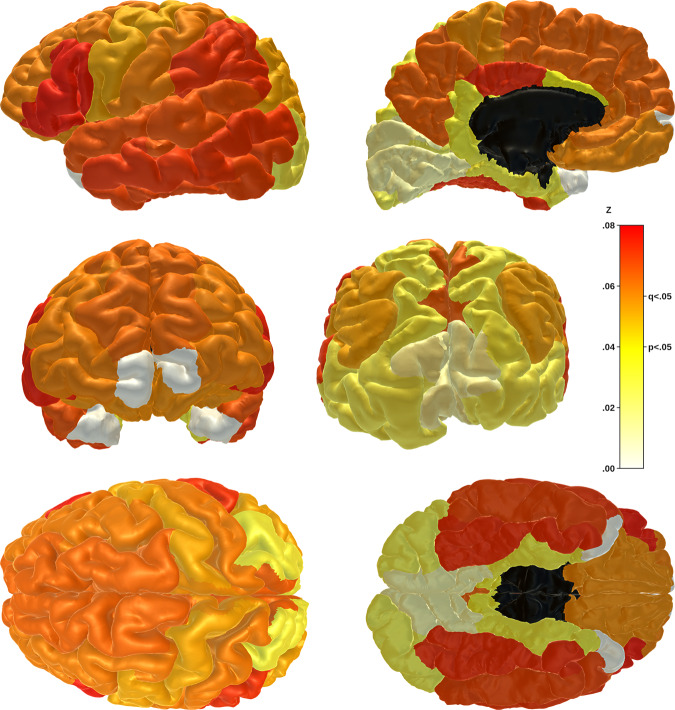
Phenotype effects on associations between lifetime depression and cortical thickness by region. *Notes*. Phenotype effects for each region represent the difference, assessed using z-tests, between the two most disparate of the β coefficients for the three phenotypes’ associations with cortical thickness of that region. Effects range from z = 0.00 (pale yellow) to z = 0.08 (bright red). Associations and effects for frontal and temporal pole regions (white) were not calculated. Z statistics for plotted results are reported in Supplementary Materials (Table S3).

As above, associations between lifetime depression and global and lobar cortical thickness were significantly affected by definition of the depression phenotype. Significant phenotype effects, assessed using z-tests of β coefficients, were also observed at the individual region/tract level for many regional cortical thicknesses (Fig. 2), as well as middle temporal gyrus volume (z = 0.054, SE = 0.016, *q* = 0.034), anterior thalamic radiation MD (z = 0.056, SE = 0.023, *q* = 0.046) and medial lemniscus MD (z = 0.072, SE = 0.020, *q* = 0.002). The strongest phenotype effects on association with cortical thickness were for inferior frontal (z = 0.076, SE = 0.024, *q* = 0.012), supramarginal (z = 0.073, SE = 0.023, *q* = 0.012) and fusiform (z = 0.073, SE = 0.023, *q* = 0.012) gyri (Fig. 3). Most phenotype effects were driven by stronger structural metric associations for Self-reported Depression in particular, while the phenotype effect for medial lemniscus MDD was driven by a stronger association with Probable MDD.

Second-level ANOVAs of individual region/tract models were performed for each of the six structural metrics to assess overall effects of depression phenotype definition. These analyses confirmed a significant overall phenotype effect on associations with lifetime depression for cortical thickness (F_2,22_ = 63.47, *p* < 0.001), but also for cortical surface area (F_2,22_ = 8.73, *p* = 0.002), cortical volume (F_2,22_ = 6.15, *p* = 0.007) and white matter FA (F_2,14_ = 4.26, *p* = 0.036).

## Discussion

We tested associations between lifetime depression phenotypes at three levels and structural neuroimaging measures, and explored effects of depression phenotype definition on these associations. Cortical thickness showed more significant results than other cortical metrics, generally associating depression with reduced cortical thickness. Self-reported Depression in particular showed robust associations with thinner cortex consistently across most regions. Cortical surface area and volume showed few significant associations after correcting for multiple comparisons, but most nominally significant results associated depression with greater cortical surface area. Multiple depression phenotypes associated significantly with reduced grey matter volume in the brainstem and ventral diencephalon, but also with greater volume of caudate nucleus and putamen. For DTI-derived metrics, depression was consistently associated with lower FA and higher MD of white matter tracts. Across both measures, results for 11 of 15 tracts remained significant after correction. Results for several tracts for each measure, primarily thalamic radiations, remained significant across the three depression phenotypes.

Effects of depression definition were generally small. Across most metrics, only volume of one cortical region and MD in two tracts showed significant phenotype definition effects after FDR correction. Cortical thickness was the exception, with 12 of 23 regions showing a significant effect of phenotype definition on depression associations. This effect was driven by the generally strong negative associations between cortical thickness and Self-reported Depression, in contrast to the weaker and less consistently negative associations with Probable Depression and CIDI-assessed MDD. Second-level analyses confirmed a much stronger effect of phenotype definition on associations between cortical thickness and depression, but also highlighted weaker overall effects of phenotype on other metrics that were less evident at the level of individual regions and tracts.

Although numerous grey matter metrics did show at least nominally significant associations with lifetime depression phenotypes, these associations were generally weaker than in previous studies [6–8, 11]. Our analyses included data for between 14,975 (Probable Depression and cortical thickness) and 26,805 (Self-reported Depression and cortical surface area) subjects, far more than most previous studies; that only relatively few associations were significant in a sample of this size is surprising. It may be attributable to studying lifetime MDD, rather than current MDD, or to the use of a population-based sample, rather than a clinical group. Most of the depression phenotypes were based on fairly limited self-report data, and likely inaccuracies in case/control classification would have diluted any genuine case effects. Furthermore, many of the nominally significant associations with cortical metrics suggested grey matter *increases* in size in MDD, also at odds with our hypotheses. However, the strongest positive associations were primarily in occipital and cingulate regions, some of which have shown MDD-related increases in size in previous studies [34, 35]. Most associations between lifetime depression and subcortical volumes were negative, as expected, although depression phenotypes were also significantly associated with greater volumes of the caudate nucleus and putamen. These positive associations seem inconsistent with previous findings [36–38], but may simply reflect lesser depression-related reduction in these subcortical structures relative to the rest of the brain.

White matter measures showed more reliable results overall, with almost 65% of associations nominally significant and over 75% of those still significant after FDR correction, consistently associating current or past depression with lower FA and higher MD. The most robust associations were for thalamic radiations, the forceps and the inferior longitudinal fasciculus. These findings are consistent with results from a previous study of a smaller subset of UK Biobank imaging participants [18], as well as studies of other cohorts [17, 19, 20]. While cortical thickness showed more significant associations with Self-reported Depression in particular, the general contrast between grey matter and white matter metrics in terms of significant results suggests changes in connectivity might be more important in depression than changes in morphology of individual regions. This would also be consistent with studies of functional data that suggest an important role of functional disconnectivity in depression [21–23].

Cai et al. [5] found that minimal phenotyping had a significant impact on depression heritability estimates and genetic associations. This seems intuitive and consistent with the assumption that minimal phenotypes, being less thorough, are also less accurate measures of depression. Conversely, our significant phenotype effects for cortical thickness were largely driven by *stronger* associations with thinner cortex for the minimal phenotype than for others. It seems therefore that minimal phenotypes may measure depression differently, rather than less accurately. If so, it may still be useful to use them alongside more thorough assessments even when these are available. White matter metrics, on the other hand, showed more consistent associations with depression across phenotypes and few significant effects of phenotype definition. This seems more comparable to Howard et al.‘s [4] results, showing similar genetic associations for different depression definitions. Grey matter metrics other than cortical thickness also showed very few significant phenotype effects, although this may simply reflect the generally lower associations between these metrics and each depression phenotype (i.e. because associations for each phenotype were so small, differences between them were also too small to detect). Overall, our results show that whether phenotype definition affects associations between depression and brain structural metrics depends on which metrics are being assessed. Again, this indicates that minimal phenotypes are not simply less accurate measures, but actually differ conceptually, which could be an important consideration for future research.

We also tested neuroimaging associations with three additional lifetime depression phenotypes (Self-reported Treatment, Recurrent Depression and ICD-diagnosed MDD), but as these were similar to the main three phenotypes presented (only with smaller sample sizes), results are included in Supplementary Materials. However, to summarise, these phenotypes also showed similar patterns of relatively weak associations with grey matter metrics and slightly stronger associations with lower white-matter microstructural integrity measures. As with our measure of Self-reported Depression, there were more significant negative associations with cortical thickness for Self-reported treatment than for other phenotypes. Results for Recurrent Depression were similar to those for Probable Depression, due to a large degree of overlap between the two, and we saw no apparent effect of multiple episodes – although this may relate to potential inaccuracies of this self reported measure. There were generally fewer significant associations with ICD-diagnosed MDD than with CIDI-assessed MDD, which may relate to differences in the case numbers since effect sizes similar or greater.

As already suggested, the imperfect criteria used to create most of the depression definitions was a limitation of this study. The available phenotypes showed associations with many brain structural metrics that were weak but still significant in our large sample, but more reliable measures may have produced stronger associations and more informative results. In particular, a structured clinical interview at the time of image acquisition would have provided a better clinical phenotype than the CIDI assessment, which was administered online at a different time. Although we used other data from the time of the imaging assessment to try to exclude CIDI-assessed cases who had only experienced depression in the time between the two assessments, these data were not as thorough as the CIDI-SF. Phenotype definition effects may also have been suppressed by the relatively low associations for each depression phenotype. Another important limitation of the study might be the population-based sample. Although this ensures generalisability (despite the UK Biobank sample being slightly healthier on average than the general population of middle-aged to older adults [39]), results may have been more robust if MDD cases had been recruited from a clinical population.

As the UK Biobank study is not specifically focused on depression, a number of covariates that would have been useful to include were not available. Furthermore, due in part to the population-based sample and some of the data collection methods, some useful covariates that were available were not complete enough to incorporate into our analyses without notably reducing sample sizes and statistical power. The limitations discussed above thereby impose a further limitation – that important covariates of lifetime depression, including contributing environmental factors, symptoms of comorbid disorders, and specific details of pharmacological or psychotherapeutic treatment, were not considered. Exploration of the effects of these and other related variables would be an important focus for a targeted study of a more thoroughly assessed clinical sample. Finally, while it was important to assess the difference between various levels of phenotyping depression as a single disorder, further research should focus on differences in brain metrics between depression subtypes, or neuroimaging associations with specific facets of depression – again, requiring a more detailed clinical study.

## Conclusion

We observed small but significant associations between lifetime depression at different levels of phenotyping and a range of structural neuroimaging measures. These were strongest for decreases in measures of white matter microstructural integrity, and for one phenotype in particular, reduced cortical thickness. The strength of associations ranged across depression phenotypes, particularly for cortical thickness, where the minimal phenotype showed *stronger* associations than other phenotypes. This suggests that depression phenotypes may differ qualitatively in terms of the construct that they measure, rather than simply differing quantitatively in accuracy of measurement. It is therefore particularly important to consider how depression is defined when conducting and interpreting research on the disorder. Despite differences between lifetime depression phenotypes, our results also provide evidence for core neuroimaging features of depression in terms of decreased integrity of thalamic radiations and association fibres across different ways of defining the disorder.

## Supplementary information

Supplement
