# Correction: Structural neuroimaging measures and lifetime depression across levels of phenotyping in UK biobank

**DOI:** 10.1038/s41398-022-02062-1

**Published:** 2022-07-14

**Authors:** Mathew A. Harris, Simon R. Cox, Laura de Nooij, Miruna C. Barbu, Mark J. Adams, Xueyi Shen, Ian J. Deary, Stephen M. Lawrie, Andrew M. McIntosh, Heather C. Whalley

**Affiliations:** 1 Division of Psychiatry, University of Edinburgh, Edinburgh, UK; 2 Department of Psychology, University of Edinburgh, Edinburgh, UK

**Keywords:** Neuroscience, Psychiatric disorders

Correction to: *Translational Psychiatry* 10.1038/s41398-022-01926-w, published online 13 April 2022

The Funding information section was missing from this article and should have read ‘This work was funded by the Wellcome Trust (Reference 104036/Z/14/Z & 220857/Z/20/Z)’.

